# 氟马替尼基础与临床研究进展

**DOI:** 10.3760/cma.j.cn121090-20231211-00304

**Published:** 2024-06

**Authors:** 磊 江, 明珍 杨

**Affiliations:** 安徽医科大学第一附属医院血液内科，安徽省公共卫生临床中心，合肥 230032 Department of Hematology, the First Affiliated Hospital of Anhui Medical University, Anhui Public Health Clinical Center, Hefei 230032, China

## Abstract

慢性髓性白血病（CML）是一种起源于多能造血干细胞的恶性血液病。伊马替尼是第一代小分子酪氨酸激酶抑制剂（TKI），使CML的治疗取得了革命性进展。氟马替尼为我国自主研发的新型口服TKI药物，可作为CML治疗的优选方案。基础研究提示氟马替尼对CML细胞株抑制作用强于伊马替尼，临床试验及真实世界中氟马替尼对CML疗效均优于伊马替尼，且氟马替尼对Ph染色体阳性急性淋巴细胞白血病、ABL特定突变CML及部分实体瘤有疗效。不良反应可控，安全可耐受。

## Abstract (en)

Chronic myelogenous leukemia（CML）is a hematological malignancy originating from the pluripotent hematopoietic stem cells. Imatinib is the first generation of small molecule tyrosine kinase inhibitors（TKI）that revolutionized the treatment of CML. Flumatinib, as a novel oral TKI that independently developed in China, which can be used as a preferred treatment for CML. Basic researches suggested that the inhibitory effect of flumatinib on CML cell lines is stronger than imatinib. Flumatinib demonstrated that it has better efficacy than imatinib on CML in clinical trials and in real world studies. Flumatinib also showed a higher potency against CML with specific mutations, Ph^+^ acute lymphoblastic leukemia and some solid tumors. The adverse events are manageable and tolerable.

自第一代酪氨酸激酶抑制剂（TKI）伊马替尼应用于临床以来，慢性髓性白血病（CML）的治疗取得了革命性进展，患者的无进展生存和总生存得到了显著延长。但仍有部分患者对伊马替尼耐药或不耐受，少部分患者疾病进展至加速期或急变期。氟马替尼作为我国自主研发的拥有自主知识产权的第二代TKI药物，被国家药监局批准用于CML慢性期（CML-CP）患者的一线治疗。此外，有报道氟马替尼在Ph阳性急性淋巴细胞白血病（Ph^+^ ALL）及伴有ABL激酶区特定位点突变的CML患者中亦有疗效。本文将氟马替尼相关基础研究及临床应用研究进展等方面进行综述。

一、氟马替尼的基础研究

氟马替尼为一种新型口服TKI药物，相较于一代TKI伊马替尼，在分子构造上，用吡啶环取代苯环的同时再导入三氟甲基，进而增强了氟马替尼与BCR-ABL激酶的结合力，提高了稳定性。三氟甲基和吡啶的吸电子基团促进了酰胺键的断裂，在体内裂解形成多种代谢产物[1]。氟马替尼在血液循环中的主要代谢产物是N-去甲基氟马替尼和酰胺水解产物，其中N-去甲基氟马替尼是主要发挥抑制酪氨酸激酶药理作用的代谢产物[2]。CYP2C8和CYP3A4是催化氟马替尼代谢的细胞色素P450酶的两个主要亚型，其中CYP3A4对氟马替尼的代谢能力强于CYP2C8。红霉素、环孢素、伏立康唑和艾沙康唑等作为CYP3A4抑制剂，能抑制氟马替尼体内代谢，提高氟马替尼的血药浓度[3]–[4]，提示临床实践中药物联用可能会增加氟马替尼的血药浓度，需要个体化调整药物剂量，防止重度骨髓抑制等不良反应的发生。此外，氟马替尼是一种脂溶性药物，高脂饮食会显著增加氟马替尼的暴露，故需空腹给药[5]。临床上，氟马替尼用于治疗CML-CP患者的标准剂量为600 mg每日1次，药代动力学参数分析表明氟马替尼暴露与剂量呈正比例的方式增加[6]，为临床联合用药及饮食结构指导氟马替尼剂量调整提供理论依据。细胞实验显示，氟马替尼（注：同HH-GV-678）呈剂量依赖性显著抑制BCR-ABL野生型细胞株和除T315I突变以外的伊马替尼耐药细胞株生长，在诱导细胞凋亡和抑制酪氨酸激酶磷酸化方面，氟马替尼对上述细胞株的作用均明显强于伊马替尼[7]。氟马替尼对包含ABL激酶区特定位点突变（如Q252H、Y253F、E255K、M351T、H396P等）的伊马替尼耐药的患者仍有疗效，但对T315I突变患者无效[8]。目前针对T315I突变的CML患者，有三代TKI普纳替尼[9]和国产的奥雷巴替尼[10]，可为T315I突变CML患者带来生存获益。

二、氟马替尼的临床研究进展

1. 氟马替尼一线治疗新诊断CML的临床试验：FESTnd研究[11]是一项多中心、随机、开放、与甲磺酸伊马替尼平行对照的Ⅲ期临床试验，394例新诊断的Ph^+^ CML-CP患者按照1∶1比例随机分配到氟马替尼组（600 mg每日1次）或伊马替尼组（400 mg每日1次）接受治疗，直至疾病进展或TKI不耐受。在主要分子学反应（MMR）率方面，用药6个月氟马替尼组为33.7％，明显高于伊马替尼组的18.3％。用药12个月氟马替尼组MMR率为52.6％，明显高于伊马替尼组的39.6％。氟马替尼组和伊马替尼组启动治疗至疗效达MMR的中位时间分别为8.5个月和11.3个月[12]。在MR4的深度分子学反应（DMR）率方面，氟马替尼组和伊马替尼组在6个月时分别为8.7％和3.6％；在12个月时分别为23.0％和11.7％。在完全细胞遗传学反应（CCyR）率方面，氟马替尼组和伊马替尼组在3个月时分别为63.1％和42.5％；在6个月时分别为85.0％和61.3％；在12个月时两组分别为91.4％和79.3％。以上结果提示：氟马替尼相较于伊马替尼治疗CML-CP患者，早期分子学反应（EMR）率更高；细胞遗传学反应率更高，反应更快、分子学反应率更高，反应更快，更深；达到MMR的时间明显缩短[11]。正是基于此项临床研究数据，中国CML诊治指南[13]推荐氟马替尼作为初诊CML-CP患者的一线治疗。氟马替尼与尼洛替尼的头对头研究提示两者用于新诊断中国CML-CP患者的疗效相当，氟马替尼组与尼洛替尼组的12个月MMR率分别为80％和78％，3个月EMR率分别为89％和85％，6个月最佳分子学反应率分别为92％和93％，两组差异无统计学意义[14]。

2. 氟马替尼治疗CML患者真实世界数据：氟马替尼在初诊CML-CP患者临床试验中取得显著疗效，在真实世界中的数据同样令人鼓舞。本研究组报道一项研究纳入5例初诊CML-CP患者，在口服氟马替尼3个月时均获得了最佳反应，其中2例患者获得MMR。有1例患者获得分子学无法检测（UMRD）并维持超过1年[15]，提示氟马替尼用于新诊断CML-CP患者疗效显著，EMR率高，有望尽早达到无治疗缓解（TFR）。包含氟马替尼在内的二代TKI治疗较伊马替尼使更多的CML患者更早获得更好的TFR[16]。另一项研究纳入80例CML-CP患者，按1∶1分为氟马替尼组和伊马替尼组，氟马替尼治疗CML患者疗效显著，MMR率高，平均BCR-ABL^IS^值低，两组不良反应率差异无统计学意义[17]。张倩等[18]报道一项研究纳入35例初诊CML患者，3个月EMR率为40％，6个月和12个月MMR率分别为43.7％和46.2％。对其他TKI药物不耐受或耐药的CML患者，氟马替尼同样显示出不错疗效，此研究纳入伊马替尼/达沙替尼不耐受患者10例，3个月EMR率为50％，6个月和12个月MMR率分别为70％和66.7％。伊马替尼/达沙替尼耐药患者11例，3个月EMR率为45.4％，6个月和12个月MMR率分别为40％和37.5％[18]。ABL激酶区突变可诱发TKI耐药，对于伊马替尼不耐受或尼洛替尼/达沙替尼不耐受且伴ABL激酶区F359V/C突变的CML患者应用氟马替尼仍可获得MR4的疗效且随访监测突变消失[19]。氟马替尼亦可克服伊马替尼耐药伴F317L突变[20]，对伊马替尼耐药伴G250E、L248V突变的CML患者亦可获得最佳反应[21]。变异的Ph易位CML患者临床少见，在9和22号染色体之外，还涉及1条或者多条染色体，氟马替尼治疗1例t（6；9；22）CML患者，3个月时达MR5，显示出优于其他TKI的疗效[22]。氟马替尼联合化疗（阿扎胞苷+维奈克拉）对于初诊CML急淋变期患者同样疗效显著，可获得CMR和CCyR的疗效[23]。

3. 氟马替尼用于Ph^+^ ALL：Ph^+^ ALL的治疗推荐多药化疗联合TKI治疗[24]。氟马替尼作为二代TKI药物，联合化疗对Ph^+^ ALL亦有显著疗效[25]。一项研究纳入25例Ph^+^ ALL患者，氟马替尼联合多药化疗28 d、3个月和6个月的CR率分别为88.00％、91.67％和90.48％；MMR率分别为68.00％、79.17％和80.95％；CMR率分别为60.00％、75.00％和80.95％[26]。另一项研究纳入了29例Ph^+^ALL患者，氟马替尼联合化疗总CR率、3个月CR率、6个月CR率分别为96.3％、87.5％和86.7％。在治疗的第1、2、3个月，MRD阴性率分别为82.6％、91.3％和95.6％[27]。中枢神经系统白血病（CNSL）是急性白血病（尤其是ALL）复发的主要根源之一，严重影响白血病的疗效。相较于达沙替尼和伊马替尼，氟马替尼在脑脊液中的浓度更高，同时对Ph^+^ ALL肿瘤细胞系SUP-B15抑制作用更强[27]。另一项研究纳入初治和复发Ph^+^ ALL患者9例，治疗28 d 4例患者获CMR，2例患者获MMR，3个月CMR率为66.7％。达沙替尼不耐受患者服用氟马替尼后，融合基因转阴并持续维持阴性状态，且一例患者T315I突变消失[28]。氟马替尼与Bcl-2抑制剂维奈克拉联用有协同作用，同时联合含地塞米松的小剂量化疗方案可为不能进行免疫靶向治疗或耐受强化疗的复发/难治Ph^+^ ALL患者提供不错疗效[29]。氟马替尼联合诱导化疗并序贯异基因造血干细胞移植对初诊Ph^+^ ALL患者具有较好疗效，且安全性良好[30]。

4. 氟马替尼用于其他疾病：氟马替尼作为新型的口服小分子TKI药物，除抑制BCR-ABL1外，还可以抑制PDGFR[31]及KIT[32]通路。相较于伊马替尼，氟马替尼对BCR-ABL1激酶的抑制作用较强，对PDGFRβ及c-KIT的抑制作用较弱，而对VEGFR、EGFR、c-Src及HER2激酶的磷酸化无作用[8]。故在PDGFR和KIT通路相关的非血液系统肿瘤中也有相应研究。超过80％的胃肠道间质瘤（GIST）患者是KIT基因突变所致，氟马替尼可以克服KIT突变，从而用于伊马替尼和Sunitinib耐药的GIST的治疗[32]。此外，氟马替尼可降低多发性骨髓瘤（MM）U266细胞株中C-MYC、HIF-1a及VEGF的表达，可能作为MM治疗的潜在新药[33]。众所周知，新型冠状病毒感染是近年最大的公共卫生事件，3a是新冠病毒SARS-CoV-2中重要的离子通道，氟马替尼可抑制3a离子通道，显示抗病毒活性，可能成为新型冠状病毒感染的潜在治疗药物[34]。但是，CML患者合并新冠病毒感染并不一定能很好地耐受氟马替尼，可能和个体差异或联合用药相关[35]。

5. 氟马替尼的不良反应：TKI用于CML患者主要的不良反应包括血液学毒性、与生活质量相关的不良反应和二代TKI特有的心肺不良反应。头对头研究显示氟马替尼血液学毒性低于伊马替尼[11]和尼洛替尼[14]，非头对头研究显示氟马替尼3～4级血液学毒性发生率明显低于二代TKI达沙替尼[36]。氟马替尼与生活质量相关的不良反应发生率更低[11]。二代TKI特有的心肺不良事件包括动脉血管栓塞事件、QTc间期延长、肺动脉高压和胸腔积液，FESTnd研究中氟马替尼组患者未发生QTc间期延长，心包积液和胸腔积液发生率极低[11]。真实世界中氟马替尼所致血液学毒性及与生活相关的不良反应发生率与临床试验报道基本一致，未监测到二代TKI特有的心肺不良反应。表明氟马替尼临床应用于CML及其他恶性疾病，不良反应可控，安全可耐受。

三、总结

氟马替尼作为新型口服小分子TKI靶向药物，用于初诊CML患者显示出确切的有效性、安全性和优于伊马替尼的细胞遗传学和分子生物学反应。与尼洛替尼临床疗效相当，3/4级不良反应发生率更低。氟马替尼联合化疗对于Ph^+^ ALL疗效确切，且耐受性良好，序贯造血干细胞移植可为Ph^+^ ALL患者带来更多获益，可作为Ph^+^ ALL患者的TKI替代选择。此外，氟马替尼可以降低MM细胞株中C-MYC等基因的表达，能有效克服GIST中某些具有激活环突变的KIT突变体的耐药性，有望成为治疗MM和GIST的潜在药物。
